# In silico prediction of variant effects: promises and limitations for precision plant breeding

**DOI:** 10.1007/s00122-025-04973-1

**Published:** 2025-07-28

**Authors:** Janek Sendrowski, Thomas Bataillon, Guillaume P. Ramstein

**Affiliations:** 1 Bioinformatics Research Center, Aarhus University, 8000 Aarhus, Denmark; 2 Center for Quantitative Genetics and Genomics, Aarhus University, 8000 Aarhus, Denmark

## Abstract

**Key message:**

Sequence-based AI models show great potential for prediction of variant effects at high resolution, but their practical value in plant breeding remains to be confirmed through rigorous validation studies.

**Abstract:**

Plant breeding has traditionally relied on phenotyping to select individuals with desirable traits—a process that is both costly and time-consuming. Increasingly, breeding strategies are shifting toward precision breeding, where causal variants are directly targeted based on their effects. To predict the effects of causal variants, in silico methods are emerging as efficient alternatives or complements to mutagenesis screens. Here, we review state-of-the-art machine learning methods for predicting variant effects in plants across both coding and noncoding regions, contrasting supervised approaches in functional genomics with unsupervised methods in comparative genomics. We discuss challenges in validating predictions, and compare these methods with traditional association and comparative genomics techniques. We argue that modern sequence models extend traditional methods by generalizing across genomic contexts, fitting a unified model across loci rather than a separate model for each locus. In doing so, they address inherent limitations of traditional quantitative and evolutionary comparative genetics techniques. However, the accuracy and generalizability of sequence models heavily depend on the training data, highlighting the need for validation experiments. We point to successful applications of sequence models, especially with protein sequences, and identify areas for further improvement, especially in modeling regulatory sequences. While not yet mature for in silico-driven precision breeding, sequence models show strong potential to become an integral part of the breeder’s toolbox.

## Introduction

Plant breeding has traditionally relied solely on phenotypic data to evaluate the breeding value of individuals. With the advent of genotyping techniques starting in the 1990s, **genetic markers** were rapidly adopted to guide the targeted transfer of genomic segments containing causal variants of interest (Ramstein et al. 2019). These techniques eventually led to genomic prediction, which jointly uses genome-wide markers and phenotypes to accelerate evaluations (Bernardo 1994; Meuwissen et al. 2001). In recent years, **precision breeding** has been emerging as a strategy that directly targets causal variants rather than broader genomic segments (Wallace et al. 2018; Ramstein et al. 2019). This approach involves molecular techniques, such as gene transformation and CRISPR-based genome editing (Gao 2021). Precision breeding has already been successfully applied in various crops to improve traits of interest, for example, in rice (Lu et al. 2021; Song et al. 2022), tomato (Rodríguez-Leal et al. 2017; Wang et al. 2021) and wheat (Zhang et al. 2019, 2021). However, in most of these applications, variants introduced by precision breeding techniques were identified through experimental mutagenesis screens, which remain relatively costly and time-consuming. In contrast, computational screens, based on in silico **prediction**, are potentially more efficient, although their resolution and accuracy may still be insufficient for routine implementation.

Methods for identifying and predicting variant effects fall into two broad research fields: **functional genomics**, where genotypes are associated with experimentally measured phenotypes; and **comparative genomics**, where fitness effects of variants are estimated by contrasting different species or populations (Ross-Ibarra et al. 2007). Both fields are well suited for applying machine learning techniques based on biological sequence data (**sequence models**), which have gained significant traction in recent years (Li et al. 2023, 2024b; Liu et al. 2024; Lam et al. 2024). We also distinguish between **supervised learning**, common in functional genomics where model training relies on experimentally labeled sequences, and **unsupervised** or **self-supervised learning** in comparative genomics, which leverages sequence variation in unlabeled data. Traditionally, functional genomics has relied on statistical associations to discover genomic segments containing variants associated with traits of interest, i.e., quantitative trait loci (QTL). QTL mapping has laid the foundation for marker-assisted and genomic selection, where desirable haplotypes are combined into improved varieties. However, such techniques lack the resolution required for precision breeding, which involves introducing targeted mutations. In functional genomics, sequence models may become useful alternatives to QTL mapping techniques (Angermueller et al. 2016; Eraslan et al. 2019). In the context of plant breeding, these models can identify or narrow down candidate causal variants for precise gene or base editing (Wallace et al. 2018; Ramstein et al. 2019).

Another important aspect of plant breeding is the purging of deleterious variants, as intense phenotypic selection during domestication and subsequent human selection may have inadvertently led to the fixation of numerous mildly deleterious mutations (Moyers et al. 2018). In comparative genomics, deleterious variants are traditionally identified by considering levels of conservation across sequence alignments spanning multiple species (Ng and Henikoff 2003; Pollard et al. 2010; Davydov et al. 2010). While alignment-based techniques have been useful for identifying impactful variants, their accuracy is constrained by limited availability of related genomes and difficulties in generating homologous alignments (Ramstein and Buckler 2022). To address the limitations of traditional alignment-based techniques, sequence models aim to predict conservation by taking into account the sequence context of the focal locus, either without incorporating alignment information (Alley et al. 2019; Rives et al. 2021; Elnaggar et al. 2022; Benegas et al. 2023; Mendoza-Revilla et al. 2024; Zhai et al. 2025) or with it (Rao et al. 2021; Benegas et al. 2025). In plant breeding, sequence models can help identify variants affecting fitness-related traits such as grain yield and biomass, (Long et al. 2022; Ramstein and Buckler 2022).

Successful precision breeding requires a detailed understanding of how the effects of genetic variants are affected by their genomic, cellular, and environmental context. This is especially challenging in regulatory regions, where most causal variants are often located (Rodgers-Melnick et al. 2016; Gullotta et al. 2023). In plants, variant effect prediction is further complicated due to large repetitive genomes, rapid functional turnover, and the relative scarcity of experimental data compared to mammals. We argue that sequence models, although conceptually similar to traditional genomic approaches, offer unique advantages for precision breeding applications, due to their ability to generalize across genomic contexts. We review state-of-the-art sequence models in functional and comparative genomics, outline their current limitations, and suggest areas of future improvement.

This review examines techniques for in silico prediction of variant effects in plant precision breeding. Whenever possible, we contrast emerging methodologies with traditional approaches and highlight their respective limitations. We first contrast supervised models in functional genomics with unsupervised models in comparative genomics, while acknowledging that the boundary between them can be blurred—for instance, when supervised models are used in comparative genomics settings. We primarily focus on plant models but reference their mammalian counterparts where equivalent plant-specific models are not yet available. After presenting conceptual and methodological differences among sequence models, we review validation procedures—ranging from cross-validation and functional enrichment analyses to direct experimental evidence—which will be critical for establishing sequence models as viable breeding tools.

## Supervised learning in functional genomics

### Predicting effects of individual variants by association testing

A common approach to detecting causal variants is association testing, which is the foundation of QTL mapping and genome-wide association studies (GWAS). In this framework, linear regression is used to estimate relationships between phenotype and genotype (i.e., allele composition at a genomic locus) in population samples usually comprising hundreds or thousands of individuals. Association testing has been the cornerstone of variant effect prediction in plant breeding because: (*i*) linear relationships between phenotype and allele count can be directly related to the additive genetic variance in a population, a parameter of immediate interest to evolutionary geneticists and breeders (Falconer and Mackay 1996); and (*ii*) linear regression models provide simple and robust estimates of variant effects on the phenotype (Legarra et al. 2021). The core statistical framework has remained essentially unchanged since its introduction in the late 1970s (Soller et al. 1976). Estimation is typically performed using a (generalized) linear regression model, accounting for potential confounders due to physical linkage (Lander and Botstein 1989; Jansen 1993) or other sources of **linkage disequilibrium** (LD), such as population structure or genetic relatedness (Yu et al. 2006; Kang et al. 2008).

Association testing is well suited for detecting variant effects on macroscopic traits directly related to breeding objectives (e.g., morpho-physiological traits, yield, disease resistance) while remaining relatively cost-effective (Li et al. 2005; Zhu et al. 2008). It has also been applied to molecular traits, such as mRNA abundance, to uncover the genetic basis of cellular and molecular phenotypes. While analyses of molecular traits facilitate variant effect detection and interpretation, they are relatively costly and typically require controlled conditions, which limits their transferability from laboratory to field settings (Poorter et al. 2016). In plants, analyses have focused on expression QTL (eQTL), which provide valuable insights into the genetic architecture of mRNA abundance, such as the proportion of gene expression variance explained by *cis*-acting variants (West et al. 2007; Kremling et al. 2018; He et al. 2022; Sun et al. 2023). However, the prohibitively high cost of molecular assays across large population samples has limited association studies of other regulatory mechanisms like chromatin accessibility (Marand et al. 2024; Zhu et al. 2024), alternative splicing (Chen et al. 2018; Zhang et al. 2024), and protein expression (Blein-Nicolas et al. 2020).

**Fig. 1:**
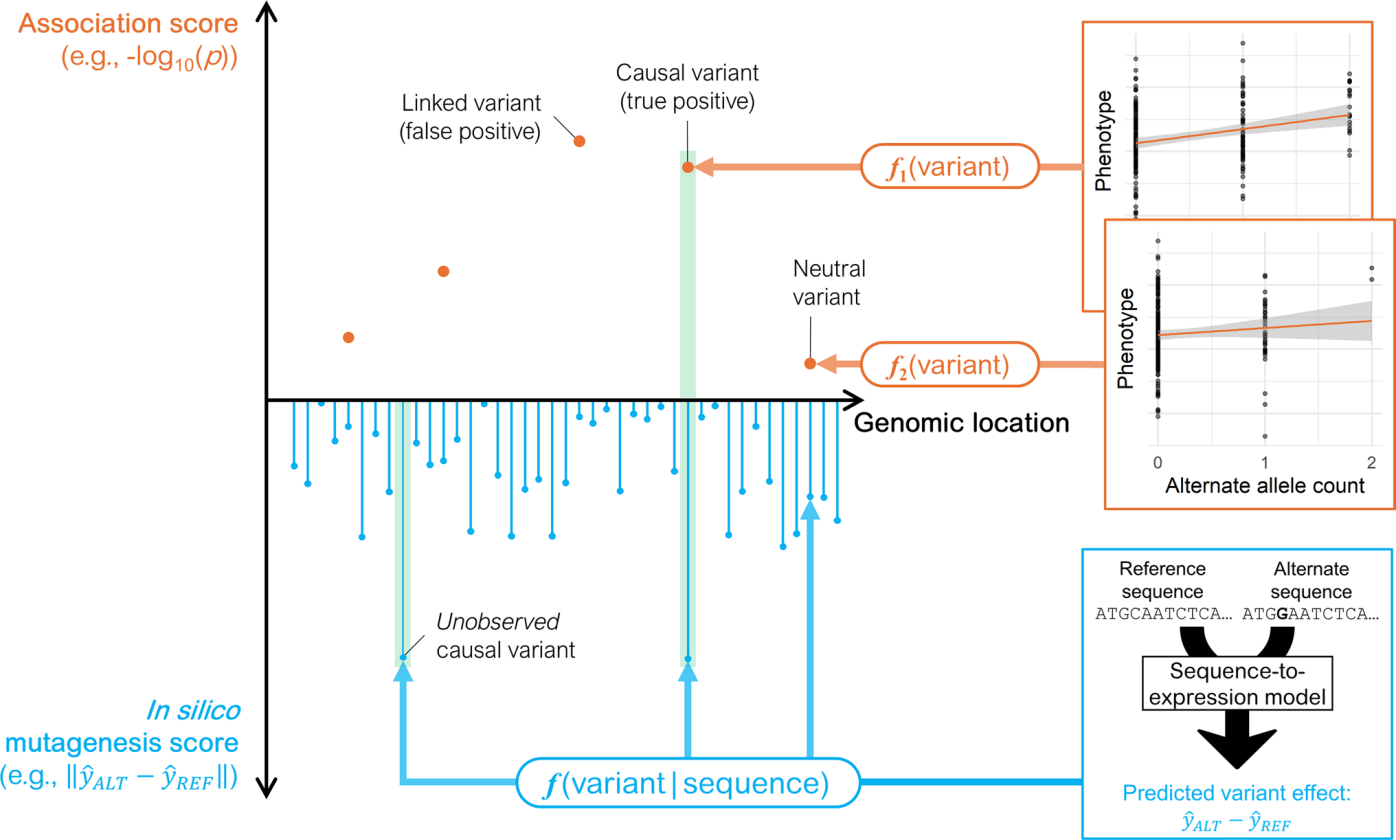
Sequence models predict variant effects through a unified function incorporating genomic context. For example, sequence-to-expression models (bottom) predict mRNA abundance based on the DNA sequence context surrounding the variant. In contrast, traditional eQTL mapping estimates variant-specific regression coefficients (top). Traditional eQTL mapping methods cannot extrapolate to unobserved variants, are prone to biases (due to LD with other variants), and can have a high sampling variance (owing to limited statistical information at each variant). In the figure, *f*(variant|sequence) represents a sequence-to-expression model generating the in silico mutagenesis score $\left\| {\hat{y}_{{{\text{ALT}}}} - \hat{y}_{{{\text{REF}}}} } \right\|$, where $\hat{y}_{{{\text{REF}}}}$ and $\hat{y}_{{{\text{ALT}}}}$ denote the predicted expression values for the reference and alternative alleles, respectively. In contrast, *f*_1_(·) and *f*_2_(·) denote two separate linear regression models, each with variant-specific regression coefficients and p-values

Association testing has inherent limitations: it estimates genotype–phenotype correlations separately for each locus, using a unique regression coefficient to estimate each allelic substitution effect (Fig. 1). In addition, estimated variant effects may be biased, inaccurate, and site-specific: (*i*) each variant is confounded by other variants in LD, such that causal variants are detected at moderate (1 kb) to low resolution (> 100 kb) (Flint-Garcia et al. 2003); (*ii*) accurate predictions require sufficient information for each variant, with statistical power being inherently low for rare variants (Sham and Purcell 2014); and (*iii*) prediction is restricted to variants observed in the study sample, so that effects cannot be extrapolated to unobserved variants.

### Modeling variant effects across genomic contexts by sequence-to-function models

**Fig. 2:**
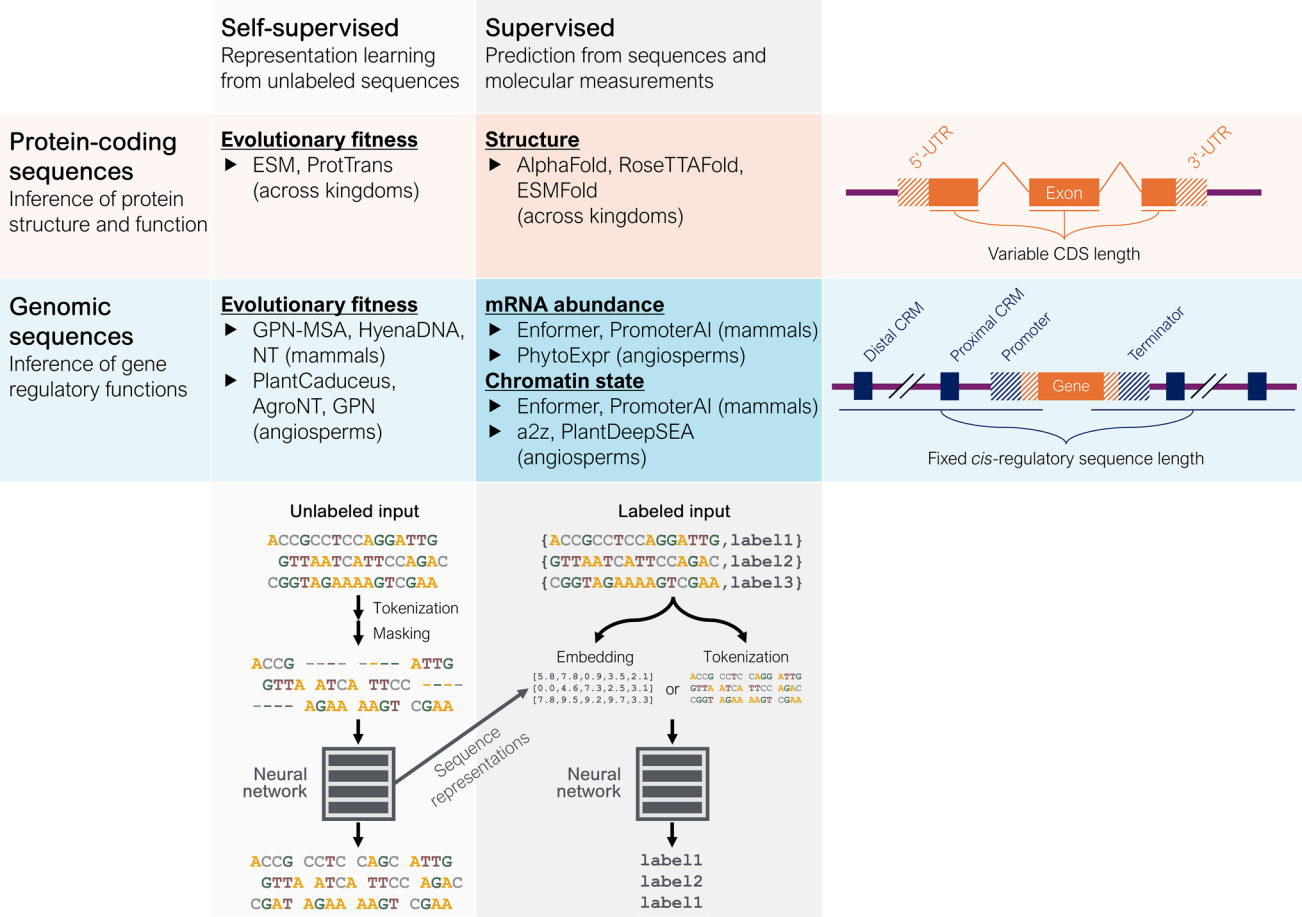
Self-supervised vs supervised, and coding vs genomic sequence models. Self-supervised/coding: Protein language models, like ESM (Rives et al. 2021) and ProtTrans (Elnaggar et al. 2022), learn representations of coding sequence grammar. Supervised/coding: Protein structure models, like AlphaFold2 (Jumper et al. 2021), RoseTTAFold (Baek et al. 2021), and ESMFold (Lin et al. 2023), are trained on experimentally determined structures. Self-supervised/genomic: Genomic language models, like GPN-MSA (Benegas et al. 2025), GPN (Benegas et al. 2023), and PlantCaduceus (Zhai et al. 2025), learn genome grammar and can either be fine-tuned for downstream tasks or used to compute conservation scores by comparing the predicted likelihood of reference and alternative alleles. Supervised/genomic: Sequence-to-expression models, like Enformer (Avsec et al. 2021a) and PhytoExpr (Li et al. 2024c), are trained on experimental measurements of mRNA abundance. Other models like a2z (Wrightsman et al. 2022) and PlantDeepSEA (Zhao et al. 2021) are trained on methylation data or measurements of chromatin accessibility like ATAC-seq data. UTR: untranslated region; CDS: protein-coding sequence; CRM: *cis*-regulatory motif

Rather than fitting a separate linear function for each locus via association testing, a single unified function may be estimated to predict variant effects based on their genomic, cellular, and environmental context (Fig. 1). Creating such an all-encompassing function is likely intractable for complex tasks, such as predicting compound traits like yield or plant height, because these traits depend on complex genomic contexts—such as sequence motifs in regulatory and coding regions at many loci—as well as specific tissues and environmental conditions. Nevertheless, **sequence-to-function models** can be trained on genomic data and applied to molecular traits in simpler tasks, such as predicting tissue-specific gene expression from *cis*-regulatory sequences or protein structure from amino acid sequences (Fig. 2). Compared to association testing which relies on linear regression, sequence-to-function models are based on different computational frameworks. Some of the first sequence-to-function models used k-mer counts and support vector machines (SVMs) to predict regulatory sequences and enhancers (Lee et al. 2011, 2015; Ghandi et al. 2014), whereas more recent models rely on neural networks to capture the complex, nonlinear effects of genetic variants (Angermueller et al. 2016; Eraslan et al. 2019). The key advantages of sequence-to-function models over association testing lie in their ability to learn relationships between any sequence and its biological function, using more data to reduce biases caused by locus-specific LD (Zhou et al. 2018). Variant effects can then be predicted through in silico **mutagenesis**, by comparing the model’s output between a reference sequence and its mutated version (Fig. 1).

Training sequence-to-function models involves several key steps: (*i*) curating a training set by selecting species, population samples, sequences, and their corresponding labels; (*ii*) defining the input format, including the sequence window around target regions and the **tokenization** method (i.e., how sequences are broken down and numerically represented); (*iii*) specifying the neural network architecture (e.g., type, number, and size of layers); and (*iv*) configuring the training procedure, including optimizer selection, learning rate adjustment, and **model performance** evaluation. Sequence labels include properties like chromatin state (e.g., chromatin accessibility and transcription factor binding), gene expression (e.g., mRNA or protein abundance), and protein structure. Among these properties, mRNA abundance is of immediate interest for breeding, since the up- and down-regulation of certain genes can be directly related to breeding values, e.g., in transcriptome-wide association studies (Li et al. 2021, 2024a; Lin et al. 2022). However, mRNA abundance is influenced by various factors—such as presence/absence and position of *cis*- and *trans*-regulatory motifs, chromatin state, and post-translational modifications—all requiring a deeper understanding of biochemical processes for accurate modeling. We focus here on sequence-to-function models that predict gene expression (**sequence-to-expression models**), which require recognizing *cis*-regulatory regions and assessing variant effects within them (Eraslan et al. 2019; Sokolova et al. 2024).

In humans and mice, the most successful sequence-to-expression models were trained on datasets for which abundant experimental data is available (ENCODE Project Consortium 2012; FANTOM Consortium and the RIKEN PMI and CLST (DGT) et al. 2014). For example, Basenji2, a **convolutional neural network** (CNN), was trained on mammalian gene expression and chromatin state data to predict gene expression levels from nucleotide sequences (Kelley et al. 2018; Kelley 2020). The state-of-the-art Enformer model is a hybrid CNN which includes **transformer** layers to account for nucleotide interactions in long sequence inputs (up to 100 kb, versus 20 kb for Basenji2) (Avsec et al. 2021a). It consistently outperformed previous models in humans and mice (Karollus et al. 2023), likely due to the **self-attention** mechanism in transformers which captures complex sequence interactions. Additional recent sequence-to-expression models with state-of-the-art performance include PromoterAI (Jaganathan et al. 2025), Borzoi (Linder et al. 2025), and ChromBPNet (Pampari et al. 2025). In plants, similar improvements have been achieved by combining transformer and convolutional layers, e.g., in the PhytoExpr model (Li et al. 2024c). However, especially for smaller datasets and models, transformers may not always show superior performance for predicting gene expression, as exemplified by a recent benchmark in maize (Wrightsman et al. 2024).

Sequence-to-function models for biological properties beyond mRNA abundance include gene expression mechanisms like alternative splicing and transcription initiation (Dudnyk et al. 2024; Xu et al. 2024), chromatin state (Zhou and Troyanskaya 2015; Avsec et al. 2021b), and protein structure (Jumper et al. 2021; Baek et al. 2021; Lin et al. 2023). Even though these models have proven useful for predicting differences between distinct genes or genomic loci, their variant effect predictions have not yet been validated convincingly. For example, in plants, it is still unclear whether sequence models predicting chromatin accessibility can be used to capture variant effects on molecular mechanisms or downstream phenotypes (Zhao et al. 2021; Wrightsman et al. 2022). Similarly, protein models such as AlphaFold2 have shown mixed results in predicting structurally disruptive variants (Buel and Walters 2022; McBride et al. 2023), likely because their training sets did not encompass small allelic differences due to point mutations (Zheng et al. 2024).

To accommodate the scarcity of experimental data in plants, sequence-to-function models may be trained on intermediate representations of input sequences. Genomic **language models**, like PlantCaduceus (Zhai et al. 2025) and AgroNT (Mendoza-Revilla et al. 2024), generate sequence representations which may be used as intermediate features, either for **fine-tuning** on downstream tasks or for training separate supervised models. However, for human data, language model features have not consistently outperformed models trained from scratch (Tang et al. 2024). In plants, the optimal strategy—direct modeling or using intermediate features—remains to be determined (see section "Modeling fitness effects across genomic contexts by biological language models" for a discussion of language models).

### Current limitations on the generalizability and resolution of sequence-to-function models

Despite advances in sequence-to-function models, association testing remains more effective for identifying impactful variants, even for well-studied traits like mRNA abundance. However, as noted above, association testing remains limited by LD-related biases and the cost and time required for sufficient statistical power. Sequence-to-function models promise to overcome these limitations, but realizing this potential will require addressing several key challenges.

#### Sequence-to-function models must be trained on relevant sources of variation

State-of-the-art sequence-to-expression models like Enformer and Basenji2 predict gene expression well across genes but perform poorly in capturing variation across human individuals (Karollus et al. 2023; Sasse et al. 2023; Huang et al. 2023). While these models can identify causative regulatory elements, they struggle to accurately predict the magnitude and the direction of variant effects on gene expression. Similar limitations have been observed in maize (Wrightsman et al. 2024), which suggests that sequence-to-function models primarily capture between-gene or cross-species variation, rather than allelic (within-gene) variation. However, these models were trained on reference genomes of one or few species and not explicitly on variant data. Including more diverse genomes or population variant data therefore has the potential to improve performance of variant effect prediction. Consistent with this, a recent study showed that fine-tuning Enformer on individual-level differences significantly improved its accuracy in predicting allelic variation in humans (Rastogi et al. 2024). However, the study also found limited generalizability to unseen genes, with prediction accuracy dropping for genes excluded from the training set. Therefore, even when trained on relevant variation, state-of-the-art sequence-to-function models may still fail to capture key determinants of variant effects. This underscores the need for fine-tuning strategies that enhance generalization to unseen variation (Jaganathan et al. 2025). In plants, it remains unclear whether similar limitations in accuracy and transferability exist. However, with more high-quality de novo assemblies and molecular data becoming available (Bayer et al. 2020; Yu et al. 2022), training sets are increasingly likely to capture informative allelic variation, i.e., differences between individuals at the same locus. In plants, predicted allelic variation may be easier to analyze than in humans due to more reproducible measurements enabled by controlled environments and genotype replication (i.e., creating genetically identical individuals through selfing or vegetative propagation).

#### Sequence-to-function models must capture the relevant determinants of variant effects

One major limitation of sequence-to-function models is the ability to learn relevant sequence features from limited training data. For example, models may struggle to account for distal *cis*-regulatory motifs (CRMs) located hundreds of base pairs upstream or downstream of the target gene. The Enformer model addresses this challenge with a considerably larger receptive field (100 kb), building on earlier models, such as Basenji2 (Kelley 2020), Xpresso (Agarwal and Shendure 2020), and Expecto (Zhou et al. 2018), which used much shorter input sequences (Avsec et al. 2021a). However, a benchmark study found that Enformer underweights distal regulators when predicting gene expression (Karollus et al. 2023). Further model development is needed, drawing on diverse sources of information—for example, composite mapping approaches like Expecto (Zhou et al. 2018), which model simple local contexts to predict epigenetic marks, then integrate these to predict gene expression.

#### Sequence-to-function models must be more computationally efficient

Many current sequence models are transformer-based and use self-attention mechanisms to learn interactions among different parts of the input sequence. Attention scales quadratically with input length, as the effects of every token on every other token are considered. This makes processing very long input sequences prohibitively expensive. Alternative methods, like sparse attention and flash attention, have been considered which are more economical (Fishman et al. 2023; Zhou et al. 2023). In addition, new network architectures such as Mamba and Hyena, scale sub-quadratically with input length, and have shown promising results in genomic language models (Nguyen et al. 2023; Zhai et al. 2025). Such models may therefore provide more efficient and accurate gene expression predictions, especially in human genomes where distal CRMs are critical. In plants, model species like *Arabidopsis thaliana* and *Brachypodium distachyon* have relatively compact *cis*-regulatory spaces, with most accessible chromatin regions located within 2000 bp of protein-coding regions (Lu et al. 2019). Therefore, these species may serve as convenient systems for evaluating sequence-to-function models, despite current limitations in capturing distal CRMs. In crop species with larger genomes (e.g., maize, wheat), addressing this limitation may be essential to improve the accuracy of predictions by sequence-to-expression models.

## Unsupervised learning in comparative genomics

### Inferring fitness effects of variants by nucleotide conservation

Traditionally, much of unsupervised learning of fitness effects in comparative genomics has centered on **conservation scores**, derived from **multiple sequence alignments** (MSAs). The intuition behind this approach is that conserved sites are under functional constraint and thus likely to be deleterious when mutated, so that the degree of conservation serves as a proxy for deleteriousness. Indeed, it has been shown that genetic load is higher in low-recombination regions of maize (Rodgers-Melnick et al. 2015), and that the use of conservation scores has the potential to improve genomic prediction of fitness-related traits like grain or biomass yield (Yang et al. 2017; Ramstein and Buckler 2022; Wu et al. 2023). Mildly deleterious variants commonly segregate in populations, and this is especially true for cultivated plants (Ramu et al. 2017; Moyers et al. 2018). Episodes of intense genetic drift may have occurred not only because of the limited number of founder genotypes during domestication, but also as an inevitable consequence of strong phenotypic selection, where effectively few genotypes contributed to breeding gene pools—allowing numerous slightly deleterious variants to accumulate alongside favorable ones. Consequently, the removal of these variants is a key objective in breeding efforts. What remains unclear is how many variants can be cleanly edited out without introducing additional unintended changes. Nevertheless, evolutionary methods may be well suited to detect such accumulated deleterious mutations. Below, we briefly outline the principles underlying these methods (Lozano et al. 2021; Kim et al. 2021; Monroe et al. 2021).

Conservation scores vary in how they estimate the number of substitutions expected under genetic drift and whether they target coding or noncoding genomic regions. Traditional methods for estimating functional constraint typically rely on MSAs, with the most advanced approaches modeling the underlying phylogeny and incorporating a probabilistic model of molecular evolution. For instance, SIFT (sorting intolerant from tolerant) scores estimate the probability that an amino acid substitution is tolerated, based on MSAs and the biochemical properties of amino acids (Ng and Henikoff 2003). GERP (genomic evolutionary rate profiling) scores are based on the number of substitutions expected under a neutral model, minus the number of substitutions observed in an MSA (Cooper et al. 2005). Despite methodological differences, Pollard et al. (2010) showed that various MSA-based genomic conservation scores have similar power to detect deleterious variants. Finally, phastCons identifies conserved regions by fitting a phylogenetic hidden Markov model to an MSA, estimating the probability that each nucleotide belongs to a conserved element (Siepel et al. 2005).

MSA-based approaches can also be combined with supervised learning. Methods such as CADD (Kircher et al. 2014), which uses a SVM, and LINSIGHT (Huang et al. 2017), which employs a linear model, also incorporate functional annotations to assess variant effects.

Traditional MSA-based conservation scores harbor inherent limitations. They are only informative if (*i*) enough mutation and recombination events have occurred between lineages to break linkage and reveal site-specific constraint (Davydov et al. 2010), (*ii*) sufficiently many genomes are aligned to ensure statistical power (Lanfear et al. 2014), and (*iii*) selection pressures are stable across the phylogeny (Huber et al. 2020). Furthermore, conservation scores rely on well-aligned regions, and are thus limited by functional turnover at orthologous sites (Rands et al. 2014; Huber et al. 2020). Functional turnover implies that capturing lineage-specific selection is challenging, and even more so for selection that is population- or environment-specific. In plants, large structural variation across species further complicates the generation of reliable MSAs (Morrell et al. 2011; Song et al. 2024).

Moreover, observed nucleotide conservation is difficult to relate directly to the actual strength of selection. For deleterious mutations, population genetics theory predicts a nonlinear relationship between nucleotide conservation (i.e., rate of rejected substitutions) and the strength of selection *S* (the selection coefficient multiplied by the effective population size): $1-\frac{4S}{1-exp(-4S)}$ (Kimura 1962; Lanfear et al. 2014). Although this theory provides a useful conceptual framework for understanding the impact of selection on nucleotide conservation, it does not apply to cross-species MSAs where its assumptions—of homogeneous, randomly mating population with a constant effective population size—are clearly violated. Although explicit mutation–selection models of codon substitution have been developed (Rodrigue et al. 2010), they have yet to be integrated into conservation score methods. This approach holds promise for disentangling the relative contributions of mutation and purifying selection to amino acid conservation, enabling more principled scoring methods.

Most importantly, conventional conservation scores take most of their information from a single focal position in the alignment, thus ignoring useful information from its haplotype context. Therefore, conventional MSA-based approaches do not generalize across sequence contexts, and cannot compute conservation scores at sites lacking an MSA. This key limitation is similar to association testing, where effect sizes are estimated independently for each variant (Fig. 1). One approach to augment information is to account for population polymorphism/divergence data, as implemented in INSIGHT (Gronau et al. 2013). However, due to the scarcity of variants in such data, information needs to be pooled across sites, and integrating this approach with conservation scores into a coherent model remains challenging. Another approach is to rely exclusively on polymorphism and divergence data. A population genetics framework can then be employed to directly quantify the strength of selection, with tools like Grapes (Galtier 2016), DFE-alpha (Keightley and Eyre-Walker 2007), or polyDFE (Tataru et al. 2017). Thus far, genome-wide summaries of polymorphism, such as the site frequency spectrum, together with divergence data, have been used to estimate the effects of mutations. To obtain region-specific estimates, the dataset can be stratified by genomic features, such as expression levels, specific regulatory regions, shared conservation scores (Chen et al. 2022; Latrille et al. 2024), or independent structural covariates like relative solvent accessibility (Moutinho et al. 2022). However, site-specific estimates remain unattainable, as individual sites lack sufficient data to support these models.

In addition to conservation scores, other sources of information can shed light on the functional or adaptive relevance of genetic variants. For instance, the likelihood that a variant is involved in local adaptation can be assessed through associations with specific environmental variables, even in the absence of phenotype data (De Mita et al. 2013; Lasky et al. 2015). Temporally spaced samples can also provide insights into recent selection by capturing changes in allele frequency over time, while accounting for the effects of genetic drift in breeding populations (Saleh et al. 2022). As full-genome data become increasingly available for crop and livestock species, methods that infer local ancestral graphs or compute summary statistics—such as the density of singletons near a focal variant—can be used to detect signals of recent directional selection (Field et al. 2016; Hartfield et al. 2021). However, the low levels of polymorphism in elite breeding populations limit the density of informative variants, thereby constraining the resolution of such approaches.

### Modeling fitness effects across genomic contexts by biological language models

Recent advancements in language models (LMs) applied to DNA and protein sequences have opened up new possibilities for predicting the fitness effects of variants using comparative genomics data (Rives et al. 2021; Benegas et al. 2023). Such models can be trained on large sets of diverse unlabeled sequences using self-supervised learning. This is typically achieved through **masked language modeling** (MLM), in which parts of the input sequence are masked and the model learns to predict the masked elements based on the surrounding context (Zhang et al. 2023; Lam et al. 2024). In doing so, LMs perform **representation learning** by extracting meaningful features from unlabeled sequences to predict the probability of nucleotides in genomic sequences or amino acid residues in protein-coding sequences as a function of the sequence context. Notably, unlike traditional alignment-based approaches like GERP and SIFT, these models do not necessarily rely on multiple sequence alignments (MSAs). LMs allow for conservation-like scoring through **zero-shot predictions** of the relative probability of variant alleles—i.e., $\text{log}\frac{\text{Pr}(\text{ALT})}{\text{Pr}(\text{REF})}$, where ALT and REF denote the alternate and reference alleles at a variant site. In addition, LMs offer alternative metrics to assess site conservation or importance, such as site entropy, and nucleotide dependencies, which quantify how nucleotides at one position influence the probability distribution of nucleotides at other positions (Tomaz da Silva et al. 2024). The learned sequence representations of LMs can also be used in **transfer learning**, where sequence encodings can serve as input to other models, or the network is fine-tuned for a specific downstream task by adjusting its weights using a much smaller dataset, labeled with experimental data (Fig. 2). The use of unlabeled sequences greatly expands the volume of training data available, which in principle allows supervised models to incorporate more information and thus improve prediction accuracy.

Significant progress in learning the grammar of coding sequences has been made by protein LMs, largely due to the high sequence conservation of coding regions across taxa, in comparison to regulatory sequences (Huber et al. 2020). LMs, such as ESM (Rives et al. 2021) and ProtTrans (Elnaggar et al. 2022), leverage the transformer architecture to predict conservation of protein residues and capture properties such as amino acid biochemistry, sequence homology, and secondary or tertiary protein structure (Bepler and Berger 2021). LMs applied to protein sequences have been shown to capture both homology and interspecies differences, performing competitively with MSA-based methods (Elnaggar et al. 2022). In humans, zero-shot predictions from protein LMs outperform conservation scores from alignment-based methods like SIFT (Brandes et al. 2023; Bromberg et al. 2024). Moreover, leveraging sequence representations from protein LMs improved performance over models that rely solely on supervised learning (Rao et al. 2019; Rives et al. 2021; Zeng et al. 2024).

Representation learning and variant effect prediction by LMs are more challenging in noncoding regions, due to considerably weaker conservation, higher functional turnover, and long-distance dependencies among *cis*-regulatory elements (Meader et al. 2010; Novák et al. 2020; Zrimec et al. 2020). Predicting regulatory variant effects is difficult yet important, as noncoding variants account for the majority of genetic variation (50% to 90%) according to statistical analyses in natural populations (Welter et al. 2014; Rodgers-Melnick et al. 2016; Watanabe et al. 2019; Gullotta et al. 2023). Consistently, a recent study pointed to the phenotypic importance of *cis*-regulatory elements in maize (Engelhorn et al. 2023). Genomic LMs, trained on large amounts of unlabeled genomic sequences, can learn sequence representations that are specific to gene regions, such as exons, introns, and promoters (Zhou et al. 2023; Nguyen et al. 2023; Benegas et al. 2023; Mendoza-Revilla et al. 2024; Dalla-Torre et al. 2024; Zhai et al. 2025). These representations can subsequently be fine-tuned for tasks, such as predicting gene expression or fine-mapping causal variants (section "Supervised learning in functional genomics").

Many genomic LMs have been introduced, differing in their training datasets, neural network architectures, and tokenization strategies. In humans, examples include NT (Dalla-Torre et al. 2024), HyenaDNA (Nguyen et al. 2023), and DNABERT-2 (Zhou et al. 2023); see (Kathail et al. 2024) for a recent review on noncoding variant effect prediction. In plants, notable models include AgroNT (Mendoza-Revilla et al. 2024), GPN (Benegas et al. 2023), and PlantCaduceus (Zhai et al. 2025). Here, we again focus on models specifically trained on plants, as genomic LMs trained on non-plant species do not transfer well to plant genomes. GPN is a convolution-based network trained on unaligned genomes of *Arabidopsis thaliana* and seven other Brassicale species, using single-nucleotide tokenization (Benegas et al. 2023). Its primary focus is conservation-based zero-shot variant effect prediction, with efforts made to debias its training set by down-sampling repetitive regions. GPN has demonstrated superior performance compared to MSA-based approaches, such as phyloP (Pollard et al. 2010) and phastCons (Siepel et al. 2005). AgroNT is a version of the NT model trained on 48 plant crop species (Mendoza-Revilla et al. 2024). Using a transformer architecture and 6-mer tokenization, it achieves competitive performance across various downstream tasks, including tissue-specific gene expression, chromatin accessibility, promoter strength, and regulatory annotation (Mendoza-Revilla et al. 2024). While AgroNT’s variant effect predictions for *Arabidopsis thaliana* are less accurate than those of GPN, it outperforms GPN in rice. This highlights a trade-off between scope and accuracy in genomic LMs, where transferability to other plant species comes at the cost of reduced accuracy in species-specific datasets. PlantCaduceus is another plant genomic LM trained on 16 angiosperm genomes using single-nucleotide tokenization (Zhai et al. 2025), and is based on the recently introduced Mamba architecture (Schiff et al. 2024). Remarkably, PlantCaduceus appeared less affected by the scope-accuracy trade-off than AgroNT. Despite being trained on diverse monocot and dicot genomes—where functional turnover is common—it appeared to outperform GPN in *A. thaliana* and other species on evolutionary constraint prediction and fine-tuned downstream tasks.

Protein and genomic LMs employ distinct strategies tailored to their respective biological contexts. Protein LMs tokenize sequences at the amino acid level, while genomic LMs use different token lengths, such as single nucleotides (GPN, PlantCaduceus) or k-mers (AgroNT). Protein LMs typically handle variable input lengths, often covering all exons of a gene. In contrast, genomic LMs are trained on genomic sequences of fixed length, often centered around a gene to capture regulatory effects. Coding regions are relatively conserved across taxa and represent only a small fraction of the genome, enabling the development of more universal protein LMs. Moreover, coding sequences from diverse organisms across kingdoms are curated for high quality and low redundancy, in datasets like UniRef (Suzek et al. 2007), providing valuable training sets for protein LMs (Alley et al. 2019; Rives et al. 2021). In comparison, training sets of genomic LMs pose greater challenges for learning biological syntax: (*i*) unlike protein sequences, genomic sequences may comprise elements from different gene regions (e.g., exons, introns, promoters), making it difficult for the model to learn a unified syntax; and *(ii*) regulatory sequences and their grammars are more variable across taxa, making more universal genomic models currently impractical.

Consequently, genomic LMs are trained on reference genomes from closely related taxa. Model architectures also differ between sequence types. State-of-the-art protein LMs typically use transformer architectures. In contrast, genomic models—facing unique challenges in capturing complex genomic patterns—leverage a variety of architectures, including transformers (AgroNT), convolutional neural networks (GPN), and more recent innovations such as Mamba (PlantCaduceus) and Hyena (HyenaDNA) (Poli et al. 2023; Gu and Dao 2023).

### Challenges and prospects for more accurate biological language models

In benchmarks for variant effect prediction, LMs have generally outperformed alignment-based approaches. However, for these models to be applicable to plant breeding, research communities in computational and experimental biology need to address critical limitations which reduce the accuracy, scope, and interpretability of variant effect predictions by biological LMs.

#### Zero-shot predictions from language models must be validated experimentally

According to functional enrichment analyses, zero-shot predictions of sequence conservation by LMs are associated with measures of fitness effect, including variant pathogenicity in humans (Brandes et al. 2023), QTL effects in Arabidopsis (Benegas et al. 2023), and allele frequencies in angiosperms (Zhai et al. 2025). Variants prioritized by predicted sequence conservation are thus likely to affect fitness-related traits, and may improve genomic selection when upweighted in genomic prediction models (Ramstein and Buckler 2022; Wu et al. 2023). However, when targeting a specific gene and tissue of interest for precision breeding, additional information is needed on the biological effects of prioritized variants. First, the gene annotation used in variant effect prediction should be ascertained. Specifically, experimental data (e.g., a gene expression atlas based on long-read RNA sequencing) should support the presence of the gene isoform used by the LM (Brandes et al. 2023). Furthermore, functional information about the gene of interest should be available, ideally from direct experimental evidence. Unfortunately, such evidence is usually lacking for the vast majority of genes in crop genomes. As a result, the limited quantity and quality of molecular genetics training data will remain a persistent bottleneck for applying predicted sequence conservation in plant breeding. To alleviate this bottleneck, computational approaches may provide additional information about the existence and function of gene isoforms (Bileschi et al. 2022; Schulz et al. 2023). However, the reliability of such surrogates is still uncertain, and to our knowledge, no research has yet evaluated pipelines for precision breeding guided by LM-based in silico predictions.

#### Protein language models must learn clade-specific fitness effects

Protein LMs are arguably the most mature technologies for variant effect predictions by sequence models, because of their phylogenetic scope (being applicable across kingdoms) and their relative accuracy compared to alignment-based techniques (Bromberg et al. 2024). Their advantages stem from their ability to learn protein syntax from large and curated datasets spanning many species (e.g., UniRef). They should effectively capture conservation patterns that are consistent across the diverse species included in the training set. In the context of plant breeding, one can then leverage these patterns to detect potentially beneficial ‘back mutations’ which revert deleterious alleles to their ancestral state (Charlesworth and Eyre-Walker 2007; Chen et al. 2022; Latrille et al. 2024). However, the species diversity present in training sets may limit the sensitivity of protein LMs and make them oblivious to clade-specific patterns of conservation. Conditional fitness effects, such as adaptive effects of variants that are specific to certain taxa, may then be missed. Focused efforts on specific clades will require additional training in less diverse sets of protein sequences. Protein LMs may then improve their power—by detecting clade-specific signals of conservation—at the expense of their precision, since they may detect spurious conservation in relatively small sets of species.

#### Optimal training strategies of genomic language models must be evaluated in standard benchmarks

Training genomic LMs presents additional challenges compared to protein LMs, particularly in (*i*) selecting relevant taxa and (*ii*) curating training sequences. Because of functional turnover in regulatory regions, training sets of genomic LMs are usually restricted to relatively small clades like mammals or angiosperms (Fig. 2). Indeed, taxon-specific pre-training has been shown to significantly improve promoter activity prediction (Fishman et al. 2023). However, it is still unclear what the optimal species diversity should be, and to what extent genomic LMs trained on diverse species can leverage that information in downstream tasks (Mendoza-Revilla et al. 2024). Crucially, it remains uncertain whether these models truly capture within-species variation rather than between-species variation (Karollus et al. 2024), suggesting that diversifying training sets by including between-individual variation may be necessary. Incorporating easily obtainable labels presents another avenue for improvement; augmenting the training data with species labels has been shown to enhance performance in species-specific gene expression tasks (Karollus et al. 2024; Li et al. 2024c). Genomic LMs may also incorporate alignment information, as demonstrated by GPN-MSA, which was trained on an MSA of diverse vertebrate genomes and achieves remarkable performance in predicting deleterious variants in humans, while requiring only a short training time (Benegas et al. 2025). The authors of GPN-MSA claim that strategically discarding the input from closely related species was crucial in avoiding overfitting to a specific clade and improving the model performance. This highlights a central challenge in training genomic LMs: selecting an optimal set of training sequences to optimize the learning of regulatory syntax. Similarly, repetitive sequences, which are abundant but less informative about regulatory variants, are often down-weighted by reducing their frequency in the training set (Benegas et al. 2023; Zhai et al. 2025). This is particularly relevant for major crop species like maize, barley, and wheat which have large genomes, highly repetitive content, and a relatively low density of functional regulatory elements (Lu et al. 2019). To identify optimal training strategies, standardized benchmarks will be critical, especially for genomic LMs for which standard curated databases like UniRef are not available (Benegas et al. 2024). Several benchmark datasets exist for human-centered genomic LMs (Marin et al. 2023; Robson and Ioannidis 2024; Feng et al. 2024). In plants, similar benchmarks (e.g., the Plant Genomic Benchmark) may facilitate the comparison of training strategies and generate useful guidelines about training genomic LMs (Mendoza-Revilla et al. 2024).

## Validation of predicted variant effects

To assess the reliability and utility of predicted variant effects, validation is crucial. Here, we discuss three main validation strategies: cross-validation, functional enrichment, and experimental validation.

### Cross-validation

**Fig. 3:**
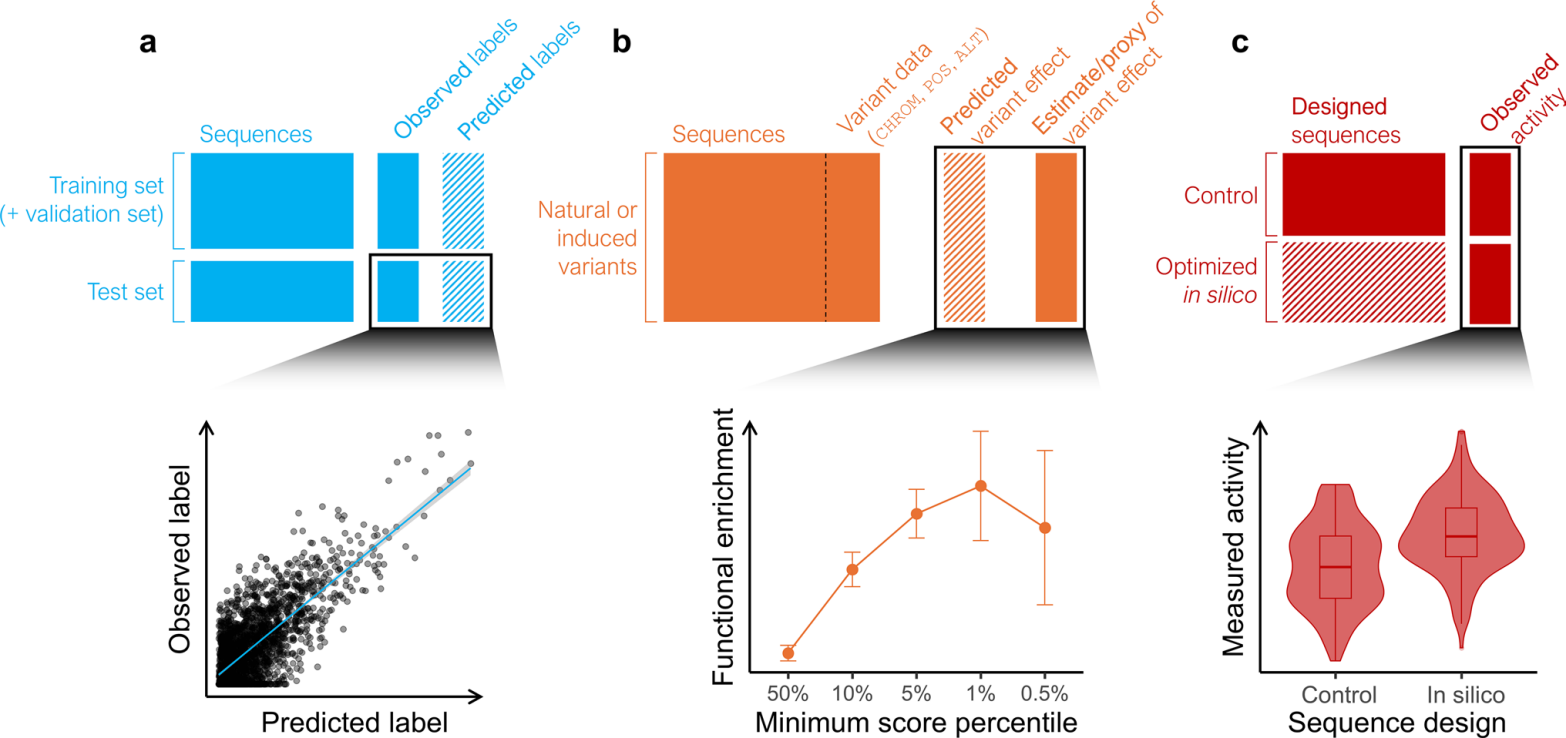
Validation of sequence models for variant effect prediction. **a** Cross-validation: model accuracy is estimated on a left-out test set, which should be designed to ensure generalizability and performance at a specific task. **b** Functional enrichment: association between predicted variant effects and independent variant effect statistics (e.g., QTL effect, allele frequency) estimated in natural or mutant populations. **c** Experimental validation: comparison of gene activity (e.g., gene expression) or organismal trait (e.g., plant physiology) between control sequences (baseline or random sequences) and sequences optimized in silico by a sequence model. Hatch patterns refer to data generated by sequence models: predicted labels, predicted variant effects, or optimized sequences

In cross-validation, model performance is evaluated on a subset of data excluded from the training process to prevent overfitting and ensure robust generalization to new data (Fig. 3a). Datasets are divided into training, validation, and test sets. The training set is used to optimize model parameters (weights), while the validation set is often used to optimize hyperparameters controlling architecture (e.g., number of hidden layers) and learning process (e.g., learning rate). The test set serves as the final evaluation dataset. Ensuring the independence of the validation and test sets from the training set is critical to minimize overfitting and ensure the model generalizes well to future datasets. Moreover, the test set should be designed to evaluate the model for its intended task. For instance, if the goal is to predict allelic differences, the test set should, whenever feasible, include examples of allelic variants, such as within-gene or within-locus variation.

Estimating a model’s ability to generalize to unseen cases is challenging, and the statistical properties of error rates based on cross-validation remain poorly understood (Bates et al. 2021). In biological sequence data, building reliable test sets is further complicated by dependencies from shared evolutionary history, such as orthologs and paralogs. Failure to account for these dependencies can lead to overfitting. To avoid splitting homologous sequences across training and test sets, Washburn et al. (2019) suggest two strategies: (*i*) gene-family–guided splitting, where distinct gene families are used for training and testing, and (*ii*) ortholog contrasts, which group orthologous genes from different species entirely within the training or test set. Similarly, Enformer groups homologous sequences into the same training, validation, and test sets by partitioning a similarity-based sequence graph (Avsec et al. 2021a), while PhytoExpr applies fivefold gene-family–guided cross-validation (Li et al. 2024c).

For protein structure prediction, standardized benchmark datasets, like CASP (Kryshtafovych et al. 2023), TS115 (Yang et al. 2018), and CB513 (Cuff and Barton 1999), enable independent evaluation of model performance, and demonstrate that predictive performance comparable to experimental evaluation could already be achieved across kingdoms for a variety of proteins. Notably, CASP prevents data leakage by testing predictions on structures not yet experimentally determined or publicly disclosed. In addition, benchmark recipes like TAPE provide evaluations for models on downstream tasks, including structure prediction, homology, and structural stability, while also defining splits for training, validation, and test sets (Rao et al. 2019). In self-supervised models, pre-training splits primarily serve to avoid overfitting, while generalization to unseen data is ensured during fine-tuning through cross-validation. For example, in the case of ESM, the dataset is randomly partitioned during pre-training, whereas, for downstream tasks such as structure prediction, cross-validation is carried out with splits based on gene family and protein fold (Rives et al. 2021).

In genomic LMs, datasets are often partitioned during pre-training either by random splitting, as in PlantCaduceus, or by reserving entire chromosomes for testing and validation, as done in GPN and AgroNT. When fine-tuning on downstream tasks, cross-validation is commonly performed with held out chromosomes. Several benchmarks have been proposed to evaluate genomic LMs (Marin et al. 2023; Robson and Ioannidis 2024; Mendoza-Revilla et al. 2024; Feng et al. 2024). Additionally, akin to the CASP competitions, a DREAM Challenge has been established to coordinate evaluation of sequence-to-expression models to predict the activity of random promoters (Rafi et al. 2024).

Generalization across individuals is critical for predicting genetic values that depend on population backgrounds. In humans, Rastogi et al. (2024) evaluated sequence-to-expression models by fine-tuning Enformer on individual genome and transcriptome data, testing its performance on unseen individuals, populations, and genes. While fine-tuning improved performance for unseen individuals, performance on unseen populations was significantly worse. This reveals limitations in capturing true variant causality, likely due to population-specific LD. Furthermore, generalization to unseen genes remained poor. The fine-tuned Enformer model largely learned linear effects of variants for gene expression prediction, similar to linear methods like FUSION (Gusev et al. 2016) and PrediXcan (Gamazon et al. 2015). Although other benchmarks report to generalize well to unseen gene families for predicting mRNA abundance (Li et al. 2024c), the findings of Rastogi et al. (2024) highlight the limitations of current sequence-based methods in generalizing to new sequences and variants, and emphasize the need for improved training strategies that better capture relevant variation.

### Functional enrichment

In addition to evaluating prediction accuracy via cross-validation, variant scores from sequence models (e.g., in silico mutagenesis scores from sequence-to-function models or zero-shot predictions from LMs) may be validated by functional enrichments, which test for statistical associations with functional variant features (Fig. 3b). Useful features for this validation include estimates or proxies of variant effects (e.g., QTL or fitness effect estimates) which are computed on a dataset independent of the model’s training set.

One proxy for evaluating the biological relevance of sequence model predictions is allele frequency in population samples. Conservation scores, which reflects evolutionary constraint, are negatively associated with allele frequency, supporting their use as indicators of selective pressure (Latrille et al. 2023). Indeed, zero-shot predictions from LMs have been shown to be more enriched for rare alleles than traditional conservation scores, indicating they better predict deleterious effects (Brandes et al. 2023; Benegas et al. 2023; Bromberg et al. 2024; Zhai et al. 2025). Variant effect predictions from sequence-to-function models have also been shown to correlate with allele frequency, even though such association arguably depends on the trait being predicted. For example, Zhou et al. (2018) showed that variants predicted to have strong effects on mRNA abundance tend to be rare, consistent with the hypothesis that deviating from optimal gene expression levels reduces fitness (Zhao et al. 2016; Kremling et al. 2018).

Another approach to validating variant effect predictions is through functional variant annotations. For example, zero-shot predictions from LMs have been shown to be enriched for QTLs affecting metabolic and morpho-physiological traits in *Arabidopsis* (Benegas et al. 2023) and mRNA abundance in humans (Dalla-Torre et al. 2024). However, QTL-based validation may be more appropriate for validating sequence-to-function models, as supervised predictions are more directly informed by biological measurements. For example, variants prioritized by their predicted effect on mRNA abundance have been validated through colocalization with eQTLs (Zhou et al. 2018; Avsec et al. 2021a; Li et al. 2024c). Importantly, observed enrichments of predicted variant effects need careful interpretation, as they may be driven by differences between genes rather than specific variant effects, e.g., through the negative correlation between gene expression and evolutionary rate (Yang et al. 2012; Zhang and Yang 2015; Ramstein and Buckler 2022). In plants, functional variant annotation remains relatively underdeveloped compared to human datasets like ClinVar or ENCODE, posing an additional challenge for reliable enrichment analyses. However, plant systems offer unique opportunities for high-resolution validation of variant effect predictions, particularly in populations where genetic differences consist of single-variant changes, rather than segregating haplotypes. Such populations may be generated through mutagenesis of independent plant lines: untargeted mutagenesis, e.g., by ethyl methanesulfonate (EMS), or mutagenesis targeted at prioritized variants, e.g., by CRISPR-based approaches (Ramstein et al. 2019).

### Experimental validation

Beyond cross-validation and functional enrichment, experimental validation provides the strongest evidence for the accuracy of predicted variant effects, and serves as a direct demonstration of their utility in molecular genetics and plant breeding. To validate the accuracy of sequence models, variants can be chosen based on their predicted impact and compared to their wild-type counterparts or randomly selected variants (Fig. 3c). To validate the impact of single-base variants *in planta*, targeted mutagenesis can be used to introduce candidate variants selected based on their predicted effects, followed by comparison with their corresponding wild types. Such mutagenesis approaches include TILLING (Henikoff et al. 2004; Tsai et al. 2011) or CRISPR-based base editing (Zhu et al. 2020; Molla et al. 2021). TILLING detects prioritized variants in libraries generated by mutagens like EMS, which preferentially induce G:C-to-A:T mutations, while CRISPR-based base editing efficiently induces G:C-to-A:T or A:T-to-G:C variants. For direct comparison of single-variant alleles, CRISPR-based editing is certainly preferable, as it introduces few off-target mutations. In contrast, TILLING requires time-consuming backcrossing to remove the hundreds or thousands of background mutations caused by mutagens (Gao 2021). However, TILLING may be more straightforward to implement in the absence of established CRISPR-based editing protocols. Moreover, large existing mutant libraries, comprising over 100,000 individuals, are readily available and can be efficiently screened—e.g., using droplet digital PCR on pooled DNA samples—to identify desirable mutant lines (Knudsen et al. 2022; Mason et al. 2024).

In plants, most experimental validations have focused on specific genes rather than individual variants. For example, some validation studies have induced loss-of-function variants to knock out candidate genes (Knudsen et al. 2022; Kan et al. 2023; Kong et al. 2023), while others modulated their expression by random promoter edits (Liu et al. 2021; Zhou et al. 2024) or by inserting constitutive promoters (Shi et al. 2017). Studies aimed at individual variants have edited candidate variants which were previously detected by experimental mutagenesis screens (Zhang et al. 2019) or QTL analyses in biparental crosses (Jiao et al. 2010; Hua et al. 2018). These studies highlight the potential of base editing for precision breeding, but also underscore the limitations of current variant discovery techniques: experimental screens are costly and so far limited to traits that are easy to assay (e.g., herbicide tolerance), while QTL analyses can pinpoint candidate variants only when segregating haplotypes differ by few variants. Sequence models offering higher throughput and resolution of predictions (e.g., by in silico mutagenesis scores) may address these limitations; however, to our knowledge, no validation studies in plants have reported on this type of approach.

*In planta* validations provide direct evidence for the utility of variant effect predictions in precision breeding, but are limited in scalability due to cost, time, and space requirements (e.g., for implementing protocols and phenotyping plants under controlled conditions). In contrast, in vitro assays can produce many transformants carrying selected sequence variants, allowing high-throughput testing at relatively low cost. A recent demonstration of this approach validated promoter and terminator strength predictions from sequence-to-function models using maize protoplasts and tobacco leaves (Jores et al. 2021; Gorjifard et al. 2024). In these in vitro assays, synthetic promoters were selected by ‘in silico evolution’ based on transcription activity predicted by a CNN. Multiple rounds of in silico evolution resulted in significantly increased transcription activity, compared to random synthetic promoters. These assays provide some of the most conclusive evidence for the accurate prediction of gene activity by sequence-to-function models. However, follow-up research is needed to determine whether these predictions can transfer to molecular activity at the whole-plant level.

## Conclusions

Biological sequence models hold significant potential for predicting the phenotypic effects of genetic variants, but their success depends on several key conditions: the input sequences must contain the major determinants influencing variant effects on the trait of interest; and training datasets must capture the relevant variability in these determinants, tailored to the specific prediction task. Furthermore, validating variant effect predictions remains limited by our ability to generate genetic variants at the required genomic resolution.

In this review, we categorized sequence models by task (self-supervised or supervised) and sequence type (protein-coding or genomic sequences). Validation studies suggest that self-supervised protein sequence models (protein LMs) are currently the most mature and suitable for precision breeding. However, rapid progress in the field is likely to expand opportunities for in silico variant discovery using DNA sequence models. As the field matures, standardized validation procedures will be essential, with varying levels of evidence provided by cross-validation, functional enrichment analysis, and experimental validation.

None of the sequence models presented here relate explicitly to phenotypes that are direct breeding goals, but only to intermediate properties such as mRNA expression or phylogenetic conservation. Despite these limitations, we see potential for sequence models in future plant breeding strategies. Potential applications include identifying candidate genes for upregulation associated with desirable traits, fine-mapping causal variants from association studies, and offering a comprehensive alternative to traditional conservation scores for identifying candidate deleterious variants for purging.

In the near future, sequence models may enhance our ability to detect causal variants influencing simple molecular traits. However, association testing will still be required to establish the link between these molecular traits and broader breeding objectives. Further advances in machine learning may eventually enable predictions for more complex molecular and physiological traits while incorporating more complex contextual factors—especially environmental data—making sequence models a key component of future breeding strategies.

## Glossary

- *Precision breeding*: a plant breeding strategy that ideally directly targets causal genetic variants based on their known effects to enhance desirable traits efficiently.
- *Genetic marker*: a measure of genetic variation (e.g., single-nucleotide polymorphism) at a genomic locus. Genetic markers usually do not capture causal variants directly but instead *tag* them through statistical associations.
- In silico* prediction*: computational methods used to predict biological effects, such as the impact of genetic variants, without physical/empirical *in planta* experimentation. It provides a cost-effective and time-efficient alternative to traditional phenotyping.
- *Functional genomics*: the field of study which aims at establishing a causal relationship between genomic variants and phenotypes. This research area includes experimental studies which assess the impact of genomic perturbations, as well as computational studies which predict the impact of genomic variants.
- *Comparative genomics*: the field of study that compares the rate of genome evolution across a set of related species with the aim to quantify and understand why levels of constraint, selection, and genome organization vary across species. Comparative genomics helps in identifying conserved genes and regulatory elements across species, shedding light on genome function and evolutionary processes.
- *Sequence model*: in the context of this paper, any machine learning model designed to process and analyze sequential data, such as DNA or amino acid sequences.
- *Supervised/unsupervised/self-supervised learning*:
  - *Supervised learning*: a machine learning approach where models are trained on labeled data, learning to map inputs to outputs by minimizing the error between its predictions and the actual labels such as mRNA abundance or chromatin state.
  - *Unsupervised learning*: a machine learning approach where models find patterns or groupings in unlabeled data, such as DNA or amino acid sequences, to identify relationships like evolutionary conservation without predefined outcomes.
  - *Self-supervised learning*: a hybrid approach where models generate their own labels from input data, particularly useful in bioinformatics where labeled data is scarce but large amounts of raw sequence data are available. It often employs methods like masked language modeling (MLM) to predict missing parts of DNA or protein sequences.
- *Linkage disequilibrium:* statistical association between genotypes at different loci due to physical linkage or co-segregation.
- *Sequence-to-function model*: a type of model used to predict functional properties of biological sequences. It maps sequence data to functional attributes, aiding in tasks like gene expression modeling or protein structure prediction.
- In silico* mutagenesis*: a computational approach that introduces mutations into a sequence and uses sequence models to predict their effects on biological functions, aiding in the identification of desirable genetic variants.
- *Tokenization*: the process of splitting a sequence, such as text or biological sequences, into smaller units (tokens) that are easier for machine learning models to process and analyze.
- *Model performance*: an evaluation of a model’s predictive capabilities across classification and continuous prediction tasks. For classification, key metrics include accuracy (proportion of correctly predicted cases) and AUC (area under the ROC curve), which summarizes the diagnostic ability of the model across all classification thresholds. The ROC curve plots the trade-off between the true positive rate (proportion of actual positives correctly identified) and the false positive rate (proportion of actual negatives incorrectly classified as positive). For continuous prediction tasks, performance is often quantified using measures of correlation, such as the Pearson correlation coefficient which evaluates the linear relationship between predicted and actual values.
- *Sequence-to-expression model*: sequence-to-function models that predict gene expression, typically measured as mRNA abundance, directly from sequence data.
- *Convolutional neural network (CNN)*: a type of neural network architecture which uses convolutional layers to extract, combine, and learn spatial features, making it highly effective for image and sequence processing tasks.
- *Transformer*: a neural network architecture designed to handle sequential data efficiently. Transformers use a self-attention mechanism to capture complex relationships between elements in a sequence, enabling parallel processing and improved context understanding.
- *Self-attention*: a key mechanism in transformers that helps models learn to focus on important relationships between positions in sequences during training. By relating each position to others, it can capture complex contextual dependencies.
- *Language model*: any probabilistic model that processes and generates language. In the context of this paper, these are neural networks trained on unlabeled sequence data using self-supervised learning. Such models are often referred to as large language models (LLMs) when trained on large amounts of data. We distinguish between protein language models and genomic language models.
- *Fine-tuning*: a process in machine learning where a pre-trained model is further trained on a specific task or dataset by adjusting its parameters. This allows the model to specialize in the new task while retaining the knowledge from its initial training.
- *Conservation score*: a metric that quantifies how conserved a nucleotide or amino acid is across an alignment. Higher conservation often indicates functional or structural importance and can serve as a proxy for the fitness impact of mutations. Examples include tools like SIFT, GERP, and phastCons.
- *Multiple sequence alignment (MSA)*: an alignment of DNA or protein sequences, often from different species, which identifies regions of similarity that may indicate functional, structural, or evolutionary relationships.
- *Masked language model (MLM)*: a self-supervised learning approach used in language models, where random tokens in a sequence are masked, and the model learns to predict the masked tokens based on their context.
- *Representation learning*: a machine learning technique where models learn meaningful representations from raw input data, such as the grammar of gene regulation, protein structure, amino acid chemistry, or DNA sequence type. This is often achieved through self-supervised learning, which utilizes large amounts of unlabeled data to extract useful features.
- *Zero-shot prediction*: a machine learning approach where a model makes predictions for tasks or categories it has not been explicitly trained on. In the context of variant effect predictions, it involves using an LM to assess the functional impact of genetic variants absent from the training data by quantifying allele conservation (i.e., $\text{log}\frac{\text{Pr}(\text{ALT})}{\text{Pr}(\text{REF})}$).
- *Transfer learning*: a machine learning technique where a model trained on one task is adapted to a related task, leveraging the knowledge gained from the first task to enhance efficiency and performance in the second. This approach is especially beneficial in situations with limited labeled data.

## Data Availability

No data is associated with this article.

## Abbreviations

CRM: *Cis*-Regulatory motif
QTL: Quantitative trait locus
eQTL: Expression quantitative trait locus
LM: Language model
MLM: Masked language modelling
LD: Linkage disequilibrium
GWAS: Genome-wide association study
CNN: Convolutional neural network
ENCODE: Encyclopedia of DNA elements
FANTOM: Functional annotation of the mammalian genome
SVM: Support vector machine
UTR: Untranslated region
CDS: Coding sequence
MSA: Multiple sequence alignment
GERP: Genomic evolutionary rate profiling
SIFT: Sorting intolerant from tolerant
ESM: Evolutionary scale modeling
GPN: Genomic pre-trained network
CASP: Critical assessment of protein structure prediction
DREAM: Dialogue on reverse engineering assessment and methods
RIKEN PMI: RIKEN Preventive Medicine and Diagnosis Innovation Program
CLST (DGT): Center for Life Science Technologies (Division of Genomic Technologies)
